# Spectrum of atypical pulmonary manifestations of COVID-19 on computed tomography

**DOI:** 10.1186/s43055-021-00448-7

**Published:** 2021-03-04

**Authors:** Balasubramanian Gurumurthy, Sudha Kiran Das, Rudresh Hiremath, Sachin Shetty, Aniketh Hiremath, Thasmai Gowda

**Affiliations:** Department of Radiology, JSS Hospital, JSS Academy of Higher Education and Research, Ramachandra Agrahara, Mysore, Karnataka 570004 India

**Keywords:** COVID-19 pneumonia, Atypical CT features, Pulmonary cysts, Bull’s eye/target sign, Halo sign, Hilar lymphadenopathy, Necrotizing pneumonia

## Abstract

**Background:**

The typical CT manifestations of COVID-19 pneumonia include ground-glass opacity (GGO) with or without consolidation and superimposed interlobular septal thickening. These are often rounded in morphology and frequently bilateral, multilobar, posterior, peripheral, and basilar in distribution. The various atypical CT features of COVID-19 are seldom described in the literature. The study aims to enumerate the atypical pulmonary CT features in patients with COVID-19 pneumonia in correlation with the disease severity.

**Results:**

A total of 298 confirmed cases of COVID-19 pneumonia with positive reverse transcription polymerase chain reaction (RT-PCR) who underwent chest CT scans were retrospectively evaluated. The cohort included 234 (78.5%) men and 64 (21.5%) women and the mean age was 53.48 ± 15.74 years. The most common presenting symptoms were fever [*n* = 197 (66.1%)] and cough [*n* = 139 (46.6%)]. Out of 298 cases of COVID-19 pneumonia, 218 cases (73.1%) showed typical CT features while 63 cases (21.1%) showed atypical CT features with concurrent classical findings and the remaining 17 cases (5.8%) were normal. Among the atypical CT features, the most common was pulmonary cysts [*n* = 27 (9%)]. The other features in the order of frequency included pleural effusion [*n* = 17 (5.7%)], nodules [*n* = 13 (4.3%)], bull’s eye/target sign[*n* = 4 (1.3%)], cavitation [*n* = 3 (1.0%)], spontaneous pneumothorax [*n* = 2 (0.6%)], hilar lymphadenopathy [*n* = 2 (0.6%)], spontaneous pneumo-mediastinum with subcutaneous emphysema [*n* = 1 (0.3%)], Halo sign [*n* = 1 (0.3%)], empyema [*n* = 1 (0.3%)] and necrotizing pneumonia with abscess [*n* = 1 (0.3%)].

**Conclusion:**

CT imaging features of COVID-19 pneumonia while in a vast majority of cases is classical, atypical diverse patterns are also encountered. A comprehensive knowledge of various atypical presentations on imaging plays an important role in the early diagnosis and management of COVID-19.

## Background

Coronavirus disease 2019 (COVID-19) is caused by a severe acute respiratory syndrome coronavirus 2 (SARS-CoV-2). COVID-19 is known to involve multiple organ systems with protean clinical and radiological manifestations, lungs being the most frequently involved [1].

The imaging changes in COVID-19 pneumonia, though classical in a majority, a small subset of it can present with diverse pulmonary findings. Based on current literature, the typical imaging features of COVID-19 pneumonia on CT include bilateral, multilobar GGOs with/without consolidation and superimposed interlobar septal thickening [2, 3]. They show a peripheral, posterior and basilar distribution [2].

Apart from anecdotal case reports, the various atypical CT features of COVID-19 are less clearly described. Anecdotal occurrence of atypical CT features has been reported till date. This study aims to enumerate the spectrum of atypical features and its incidence as observed at our institute in correlation with disease severity.

## Methods

### Study design and data collection

This was a retrospective observational study conducted during the period from 1 July till 31 October 2020. The study included reverse-transcriptase polymerase chain reaction (RT-PCR) confirmed 298 COVID-19 pneumonia patients who were admitted to the hospital and underwent chest HRCT scan. Images were evaluated using the institutional Picture Archiving and Communication Systems (PACS) database system to assess the various imaging features in COVID-19 pneumonia cases. This study was approved by the Institutional Ethics committee.

### Selection criteria

#### Inclusion criteria

Reverse-transcriptase polymerase chain reaction (RT-PCR) confirmed COVID-19 pneumonia patients who underwent chest HRCT scans were considered.

#### Exclusion criteria

- Patients with negative RT-PCR results.
- Patients discharged to another facility during the course of illness.

### CT imaging protocol

CT was performed using a 128-slice MDCT scanner (Ingenuity core 128 v3.5.7.25001; Philips healthcare). Patients were placed in a supine position with single breath hold. Scanning parameters were scan direction (craniocaudally), tube voltage (120KV), tube current (250 mA), slice collimation (64 × 0.625 mm), width (0.625 × 0.625 mm), pitch (1), rotation time (0.5 s), and scan time (12.06 s). Images were reconstructed with a slice thickness of 0.5 or 1.5 mm and an interval of 0.5 or 1.5 mm, respectively.

### CT imaging interpretation

The images were evaluated by two senior professors and a senior resident in radiology with experience of 17 years, 15 years, and 4 years, respectively. The evaluators independently assessed the CT features using both axial CT images and multiplanar reconstruction images. The scans were first assessed whether negative or positive for typical findings of COVID19 pneumonia (bilateral, multilobar, posterior peripheral ground glass opacities) as defined by the RSNA Consensus statement [4, 5]. Later the various atypical CT imaging features were noted. Severity was assessed using CT severity score (total score out of 25) and categorized into mild (score-< 7), moderate (score 7–18) and severe (score > 18) as done by Saeed G A et al. [6]. The two senior professors were blinded to the outcome of the cases.

### Statistical analysis

The analysis was performed using SPSS 21.0. Descriptive statistics of patients’ demographics and clinical results were reported as numbers and relative frequencies. The mean differences between the groups with typical and atypical CT features were subjected to Mann–Whitney *U* test to check for the statistical significance. The Spearman correlation coefficient test was used to investigate the correlation between the groups with typical/atypical CT features and mortality and also was used to investigate the correlation between the age and CT severity score among the patients with atypical CT features.

## Results

A total of 298 reverse-transcriptase polymerase chain reaction (RT-PCR) confirmed COVID-19 pneumonia patients admitted between 1 July and 31 October 2020 were retrospectively evaluated. The demographics pertaining to age, gender, presenting symptoms, presence of comorbidities/risk factors, CT severity score, and disease outcome (alive or died) are as below.

The mean age in our cohort was 53.48 ± 15.74 years [range 9–90 years]. The age was further classified into 9 groups: (0–10), (11–20), (21–30), (31–40), (41–50), (51–60), (61–70), (71–80), and (81–90) as depicted in the Table 1. The age group (51–60) had highest cases with total of 79 cases (26.5%) There was predilection for males 234 (78.5%) when compared to female 64 (21.5%).

**Table 1:** Age distribution of the cohort

Age (in years)	Total number (out of 298)	Percentage (%)
0–10	1	0.4
11–20	1	0.4
21–30	27	9
31–40	39	13
41–50	51	17.2
51–60	79	26.5
61–70	55	18.5
71–80	36	12
81–90	9	3

The most common presenting symptoms were fever [*n* = 197 (66.1%)] and cough [*n* = 139 (46.6%)]. Other symptoms were dyspnea, generalized weakness, myalgia and others with frequencies as depicted in the Table 2.

**Table 2:** Presenting symptoms of patients with COVID-19 pneumonia

Symptom	Total number	Percentage (out of 298)
Fever	197	66.1
Cough	139	46.6
Dyspnea	83	27.8
Generalized weakness	32	10.7
Myalgia	29	9.7
Hemoptysis	2	0.6
URTI symptoms	18	6.0
Diarrhea	16	5.3
Vomiting	8	2.6
Easy fatigability	33	11.0
Palpitation	1	0.3
Pain abdomen	8	2.6
Chest pain	12	4.0
Headache	10	3.5
Loss of smell/anosmia	5	1.6
Loss of taste	2	0.6
Hemiparesis	3	1.0
Loss of appetite	16	5.3

The comorbidities/risk factors considered were hypertension, diabetes mellitus, cerebrovascular diseases, and others as depicted in the Table 3. Comorbidities/risk factors were found in 161/298 patients (54.0%). The most common associations were diabetes mellitus [*n* = 100(33.5%)] and hypertension [*n* = 94(31.5%].

**Table 3:** Comorbidities/risk factors of patients with COVID-19 pneumonia

Co-morbidities	Total number	Percentage (out of 298)
Diabetes	100	33.5
Hypertension	94	31.5
Cardiovascular disease	23	7.7
Cerebrovascular disease	4	1.3
Chronic kidney disease	5	1.6
Hypothyroidism	9	3.0
Chronic pulmonary disease	14	4.6
Immunocompromised	2	0.6
Others	3	1.0

Out of 298 cases of COVID-19 pneumonia, 218 cases (73.1%) showed typical CT features while 63 cases (21.1%) showed atypical CT features with concurrent classical findings and the remaining 17 cases (5.8%) were normal. Among the atypical CT imaging features, pulmonary cysts were the most common feature in our study. Other various atypical imaging features with their incidences in our study are as depicted in the Table 4.

**Table 4:** Incidences of the atypical CT findings of COVID-19 among the study group

SL no.	Atypical CT imaging features	Number of cases	Percentage (out of 298 cases)
1.	Pulmonary cysts	27	9.0
2.	Pleural effusion	17	5.7
3.	Nodules	13	4.3
4.	Bull’s eye/target sign	4	1.3
5.	Cavitation	3	1.0
6.	Spontaneous pneumothorax	2	0.6
7.	Hilar lymphadenopathy	2	0.6
8.	Spontaneous pneumo-mediastinum, subcutaneous emphysema	1	0.3
9.	Halo sign	1	0.3
10	Empyema	1	0.3
11.	Necrotizing pneumonia with abscess	1	0.3

In our cohort, the pulmonary cysts on HRCT were well defined, thin walled (2–4 mm) and of size < 2.5 cm. The most common pattern of distribution of pulmonary cysts was peripheral lung distribution [*n* = 23(85.2%)]. Other patterns were random distribution [*n* = 3(11.1%)] and perihilar distribution [*n* = 1(3.7%)]. They showed lower lobe predominance (*n* = 15) when compared to upper lobe (*n* = 9) and middle lobe (*n* = 2).

The nodules on HRCT showed peripheral subpleural distribution [*n* = 12]. Only one case showed centrilobular distribution with tree in bud pattern. Both solid (*n* = 6) and GGO (*n* = 7) nodules were equally seen. No difference in the lobar predominance [upper lobe (*n* = 6) and lower lobe (*n* = 7)].

Out of 17 cases of pleural effusion, 13 cases showed bilateral effusion. A total of 15 cases showed mild effusion and two showed moderate effusion. In 10 cases, there was no presence of other co-existent diseases while 7 cases had co-existent diseases.

All four cases with the bull’s eye sign/target sign were all peripherally located in the lower lobes.

All cases with cavitation (*n* = 3) showed peripheral distribution with two cases involving the lower lobe and one in the upper lobe.

Two cases showed bilaterally enlarged hilar group of lymph nodes with short axis diameter of > 10 mm with more on right side compared to left. Loss of hilar fat pad was noted in both the case.

### Correlation between atypical CT imaging features with age and severity

Significant positive correlation between CT severity score among atypical group and age was observed in our study (*ρ* = 0.343 and *p* = 0.006), indicating that with increase in age there was increase in the CT severity score and also of atypical CT features. Out of 63 patients with atypical features, CT severity score were as depicted in Table 5.

**Table 5:** Atypical features of COVID-19 cases and their CT severity score

CT severity score (out of 25)	Total number(out of 63)	Percentage (%)
Mild (< 7)	26	41.26
Moderate (8–17)	25	39.68
Severe (> 18)	12	19.04

However, there was no statistically significant correlation (*p* > 0.05) between typical or atypical CT features and the severity of the disease (CT severity score).

In terms of clinical outcome, 289 patients (97%) were discharged after clinical improvement and 9 patients (3%) died in hospital at the time of the study. Out of 9 demises, CT severity was of moderate (score 8–17) and severe category (score > 18). All cases were aged above the mean of 53.4 years and majority was associated with comorbidities (*n* = 8). Males [*n* = 7(78%)] were affected more compared to females [*n* = 2(22%)]. However, no statistically significant association between typical or atypical CT features and mortality was noted (*p* > 0.05).

## Discussion

Coronavirus disease 2019 (COVID-19) is caused by severe acute respiratory syndrome coronavirus 2 (SARS-CoV-2). This has rapidly resulted in a worldwide pandemic with significant increase in morbidity and mortality [1]. The imaging changes in COVID-19 pneumonia are diverse with the various atypical CT features being less clearly described. The study conducted herein explains the atypical CT features in COVID-19 pneumonia.

In our study, the most common presenting symptoms were fever and cough consistent with the study by Shi H et al. [7]. There was predilection towards males (78.5%) as seen in the study done by Huang C et al. [8]. The other predisposing conditions in our study were elderly patients (mean 53.4 years) and comorbidities which are consistent with the study by Shi H et al. [7]. The mortality rate in our study was 3%. All the deaths occurred in elderly patients who had comorbidities, consistent with previous reports (8) and had moderate-severe CT severity score. Hence, advanced age, male sex, presence of comorbidities and higher CT severity score might be risk factors for poor prognosis.

Based on current literature, the typical imaging features of COVID-19 pneumonia on CT include bilateral, multilobar GGOs with/without consolidation, and superimposed interlobar septal thickening [2, 3]. They show a peripheral, posterior, and basilar distribution [2]. In our study, majority of the patients (73.1%) showed typical CT features and only 21.1% patients showed atypical CT features with concurrent above classical findings. Among the atypical CT features, the most common was pulmonary cysts. The other features in the order of frequency included pleural effusion, nodules, bull’s eye/target sign, cavitation, spontaneous pneumothorax, hilar lymphadenopathy, spontaneous pneumo-mediastinum with subcutaneous emphysema, halo sign, empyema, and necrotizing pneumonia with abscess.

**Fig. 1:**
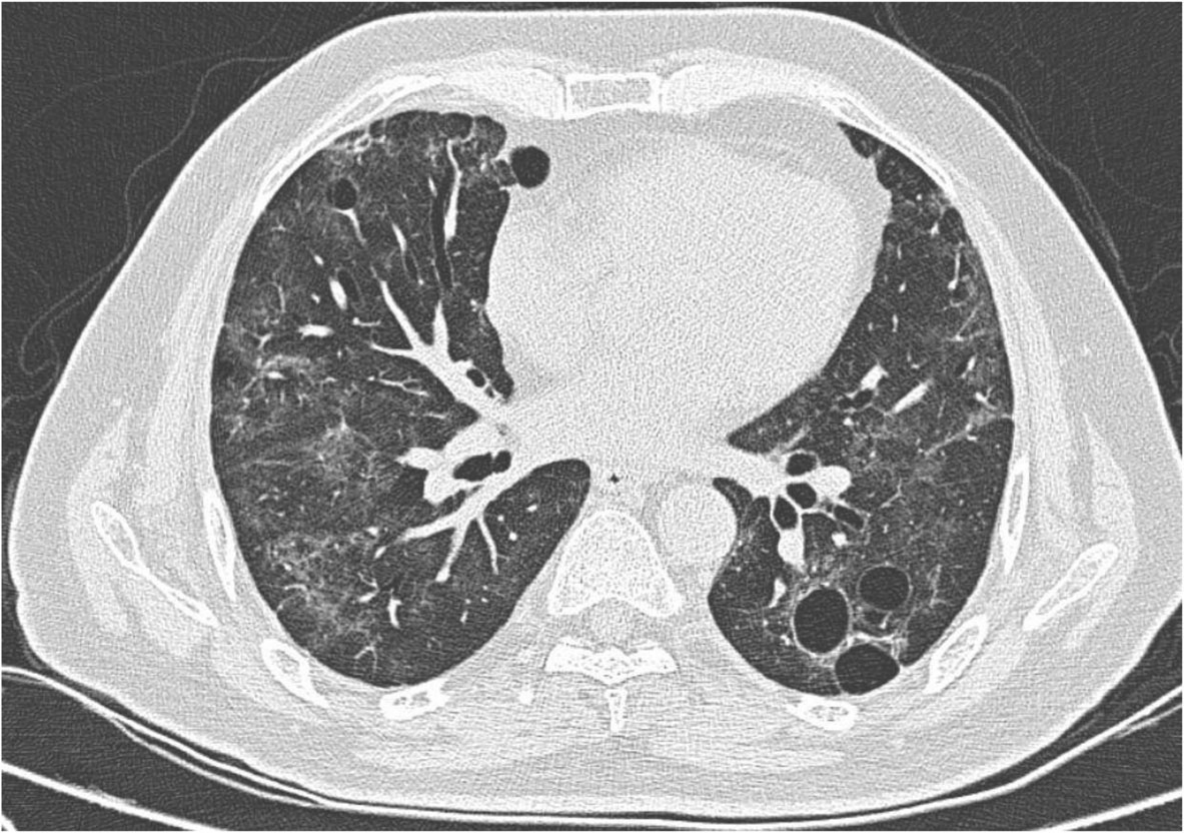
Axial CT thorax of a 60-year-old COVID pneumonia patient showing multiple thin-walled air-filled cystic lesion in the bilateral lower lobes. Areas of GGOs with interlobular septal thickening are also seen

The incidence of pulmonary cysts in our study was 9.0% which is consistent with the study by Shi H et al. [7]. Recent studies speculate that the pulmonary cystic change in COVID-19 might be secondary to ischemic parenchymal damage, lung fibrosis and low lung compliance [9]. Another explanation is blockage of the bronchioles by mucus and mucus plugs followed by the over-inflation of the alveoli and resultant rupturing of the alveolar septum with subsequent formation of small cysts [10]. None of the patients in our study were on mechanical ventilation and hence ruling out barotrauma induced cystic changes [9]. The peripheral subpleural cysts are prone to rupture causing pneumothorax. Hence, in COVID-19 pneumonia, prominent identification of pulmonary cysts and close monitoring for complications are required. Figure 1 shows pulmonary cysts in a case of COVID-19 pneumonia.

**Fig. 2:**
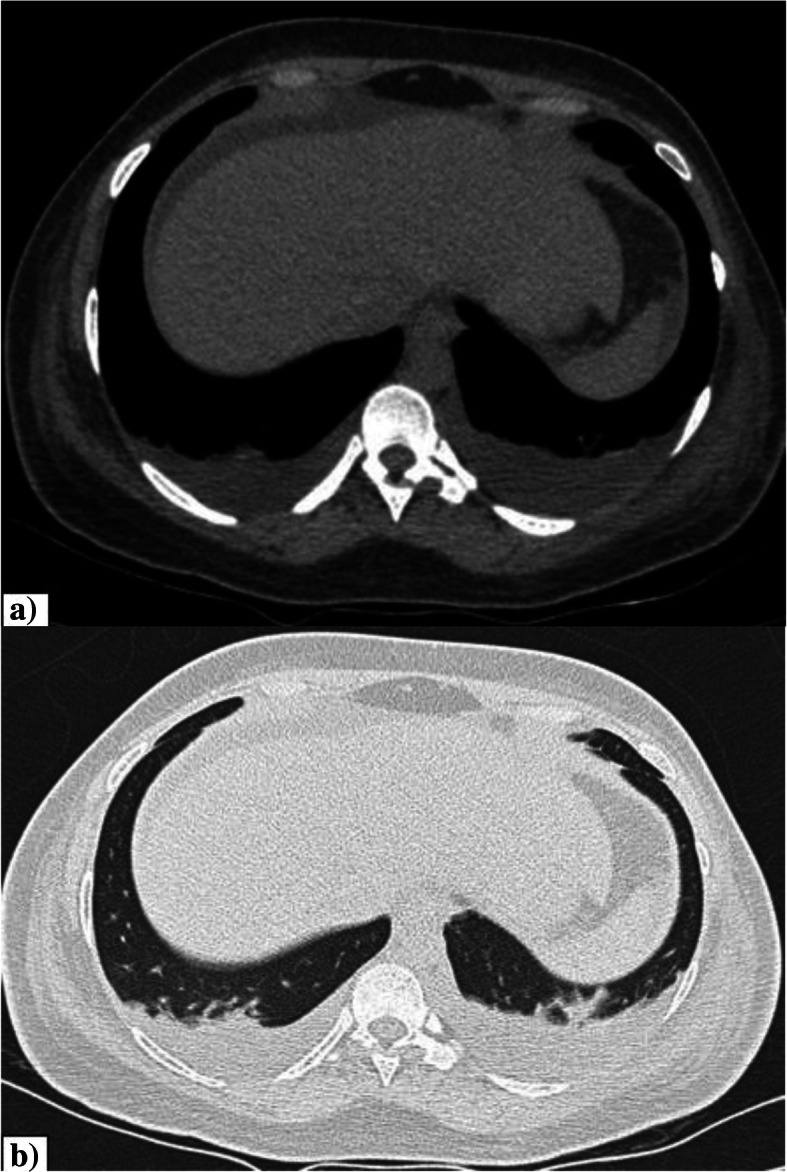
Pleural effusion. **a**, **b** Axial CT thorax (mediastinal window and lung window) of a 38-year-old COVID pneumonia patient showing bilateral pleural effusion with underlying sub-segmental collapse

The incidence of pleural effusion in our study was 5.7%. However the incidence of pleural effusion in COVID-19 has been reported to be varying as per the available literature [11]. According to the study by Shi et al., the prevalence of pleural effusion varies depending on the stage of the disease, with a reported prevalence of 13% in the third week after onset of symptoms [7]. Pleural effusion may also be predictive of worse prognosis [11]. The presence or absence of underlying medical conditions, study setting, disease stage, and concurrent superimposed bacterial pneumonia are to be considered in order to comment on the prevalence and etiology of pleural effusion in COVID-19 infection [12]. The presence of effusion in cases with no concurrent comorbidities can possibly be attributed to COVID-19 infection or superadded bacterial infection. Figure 2 shows bilateral pleural effusion in a case of COVID-19 pneumonia.

**Fig. 3:**
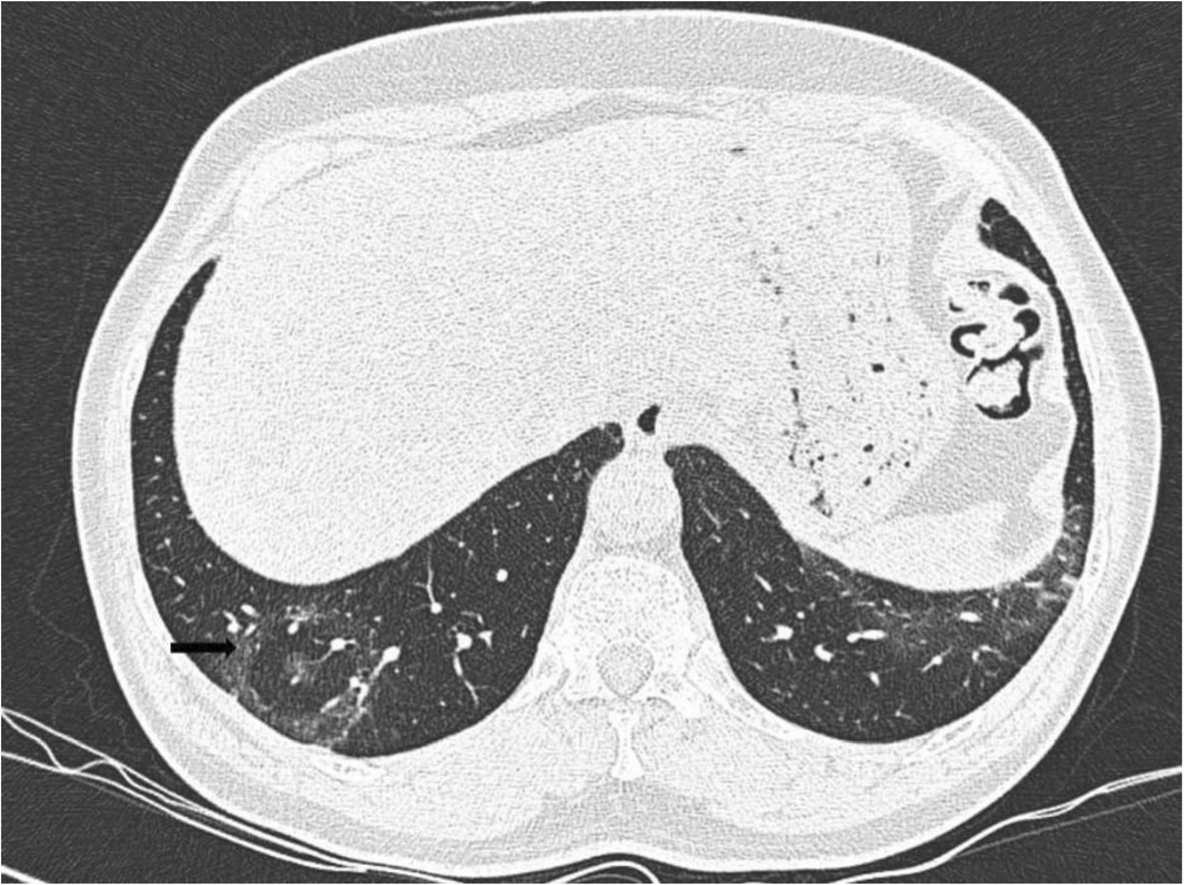
Bull’s eye sign. Axial CT thorax of a 55-year-old COVID pneumonia patient showing central ground glass opacity surrounded by an inner ring of air and an outer ring of ground glass in the right lower lobe

The bull’s eye/target sign consists of central ground glass opacity surrounded by an inner ring of air and an outer ring of ground glass as shown in Fig. 3. It is presumed that bull’s eye sign may be a variant of the reverse halo sign [13]. In our study, bull’s eye sign accounted to 1.3% of the cases. Only few case reports on COVID-19 with bull’s eye sign has been made in the literature [13–15]. It has been theorized that they represent regions of organizing pneumonia, with perilobular involvement and a tendency to be located peripherally within the lung parenchyma [13]. The bull’s eye sign/target sign in the presented cases likewise were all peripherally located in the lower lobes [13].

**Fig. 4:**
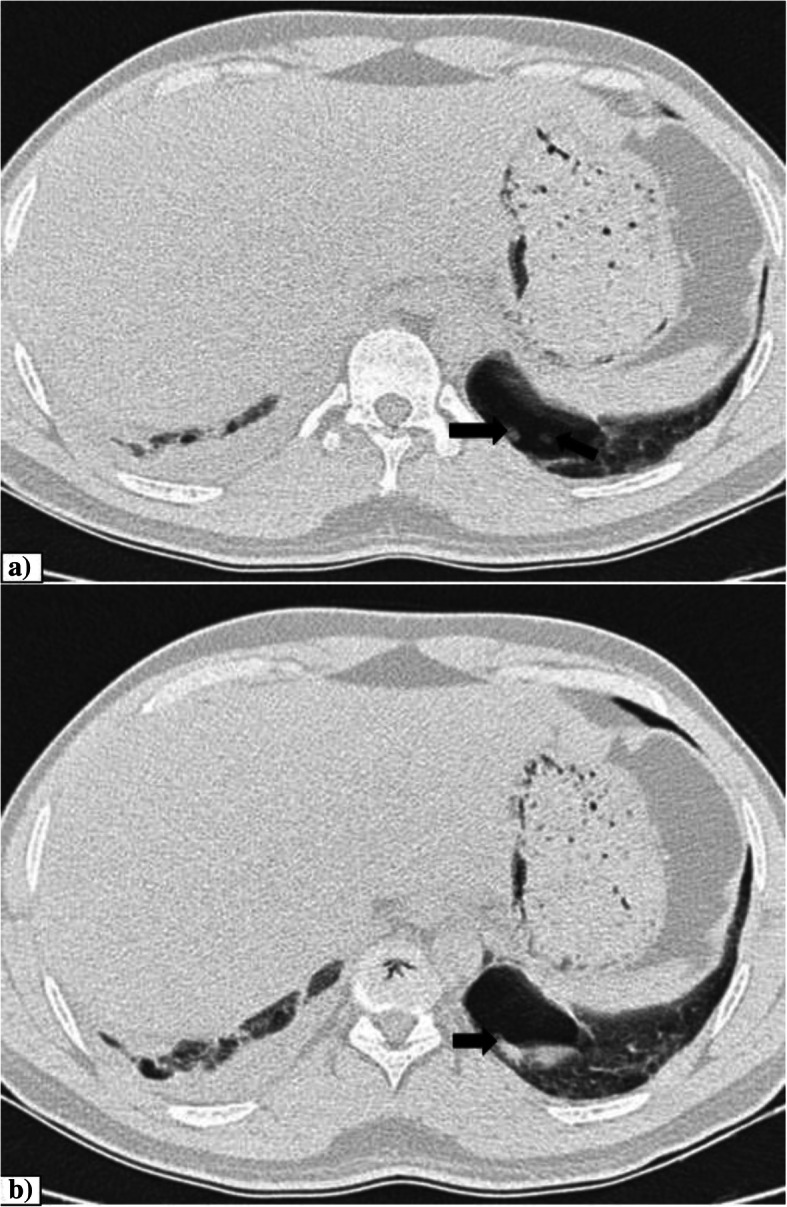
Pulmonary cavity with clots. **a**, **b** Contiguous sections of axial CT thorax of a 35-years-old COVID pneumonia patient who presented with hemoptysis, showing thin wall cavity in the posterior basal segment of left lobe with soft tissue component of hemorrhagic density as indicated by the arrows. Right pleural effusion is also seen

Lung cavitation due to COVID-19 pneumonia is an uncommon finding which usually is seen in the late stage [16, 17]. The incidence in our study was 1%. There are few reports of intrapulmonary cavities of COVID-19-infection [11, 16, 18, 19]. However majority of other reviews showed no cavitation in their study [7, 20–23]. The cavitation may be related to diffuse alveolar damage, intra-alveolar hemorrhage, and necrosis of parenchymal cells based on prior autopsy reports [24, 25]. Common causes of cavitary lung lesions must be investigated appropriately in all patients [26]. In our study, there was no laboratory evidence supporting bacterial infections. Hence, the possibility of COVID-19 independently resulting in cavitation is to be considered. Close monitoring is required for complications like hemorrhage within the cavity, rupture of peripheral cavity resulting in pneumothorax, and superadded bacterial infection resulting in an abscess. Figure 4 shows pulmonary cavity with clots in a case who presented with hemoptysis.

**Fig. 5:**
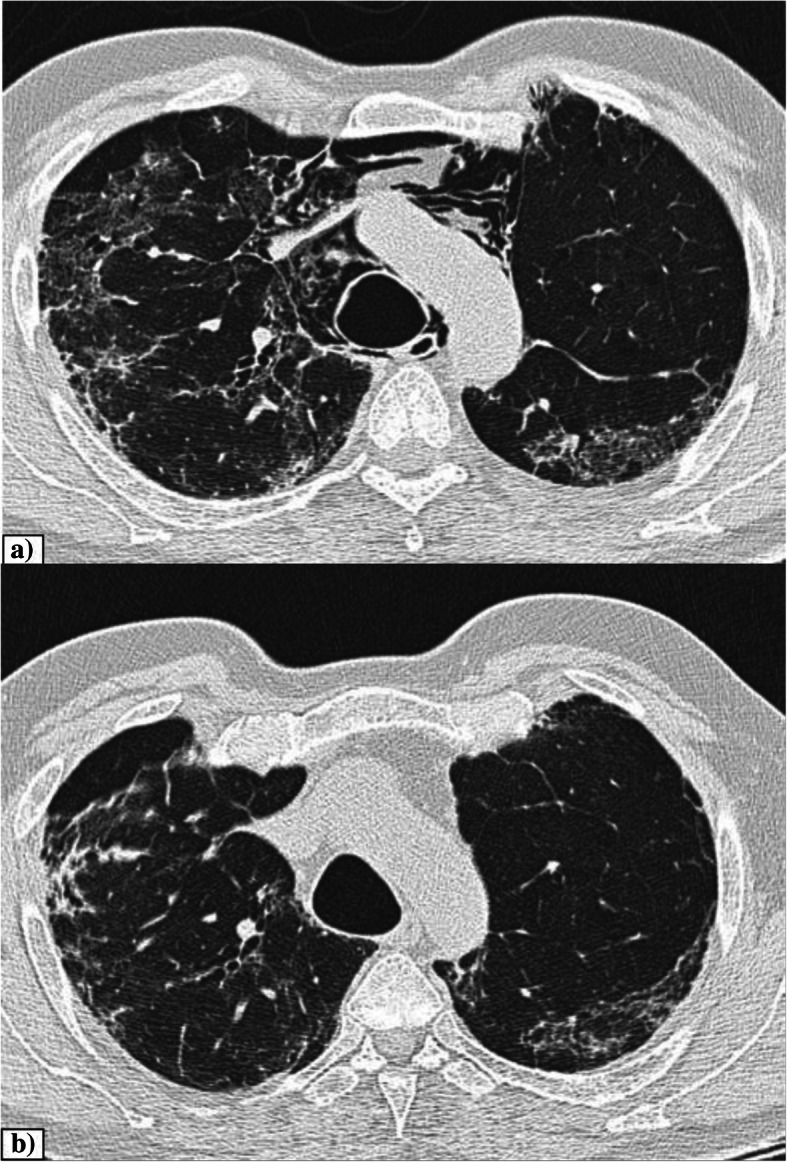
Spontaneous pneumomediastinum. **a** Axial CT thorax of a 58-year-old COVID pneumonia patient showing pneumomediastinum in the pre-vascular space and around trachea and esophagus. Visualized lung shows peripheral GGOs with interlobular septal thickening. **b** Complete resolution of pneumo-mediastinum after 10 days

Spontaneous pneumomediastinum (SPM) refers to the presence of air in the mediastinum occurring in the absence of traumatic or an iatrogenic origin [2, 27]. In current limited research, only few case reports of SPM in COVID-19 have been made [2, 28–30]. The incidence of SPM in our study was 0.3% and isolated spontaneous pneumothorax was 0.6%. Chen N et al. showed incidence of isolated spontaneous pneumothorax of 1% in COVID-19 patients [20]. It is believed that the possible causes of SPM in COVID-19 were similar to those in SARS showing severe diffuse alveolar damage. This diffuse alveolar damage results in alveolar rupture which can be further precipitated by high interalveolar pressure caused by factors like artificial ventilation, coughing or straining. This results in air migration into the mediastinum through the Macklin effect [2, 31–33]. The SPM can lead to other complications such as pneumothorax, extensive subcutaneous emphysema, and an uncommon complication of lung infections [2]. In our study, none of the cases were mechanically ventilated at the time of initial CT imaging. One case with emphysematous changes showed SPM with pneumothorax and extensive subcutaneous emphysema as shown in the Fig. 5, which we believe would have occurred due to progression of pre-existing lung lesions resulting in rupture of subpleural bulla or secondary to alveolar rupture as described above. Isolated spontaneous pneumothorax in our study might be secondary to rupture of subpleural pulmonary cysts or due to alveolar rupture.

**Fig. 6:**
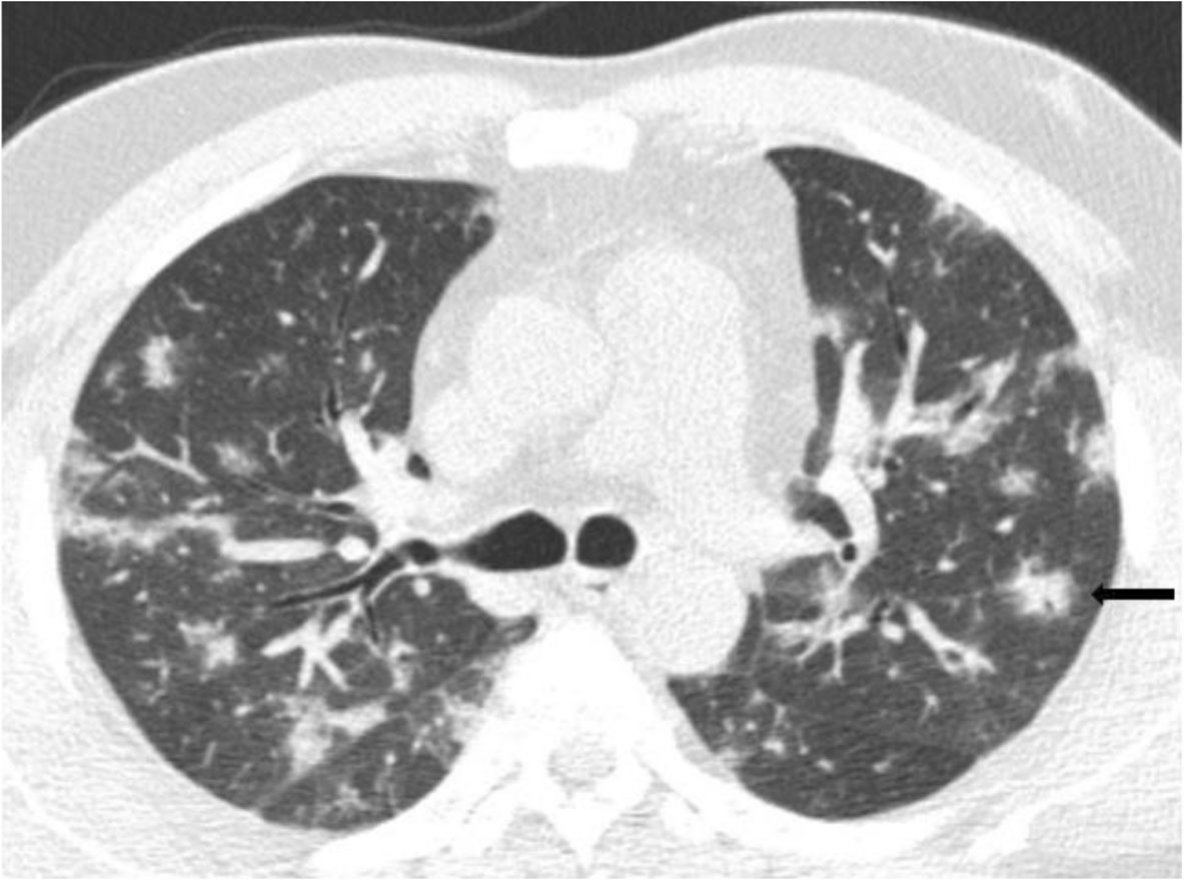
Halo sign. Axial CT thorax of a 40-year-old COVID pneumonia patient showing multiple bilateral patchy consolidations with surrounding ground-glass opacities as indicated by the arrow

The halo sign represents area of consolidation/pulmonary nodule/mass surrounded by ground-glass opacity [34–36] as shown in the Fig. 6. The incidence of halo sign in our study was 0.3%. In current limited research, only few cases reports on the halo sign has been made in COVID-19 [11, 37, 38]. Based on the pathological findings as seen in some recent studies, extensive thrombotic damage of the pulmonary microcirculation can explain the “halo sign” of consolidations [34].

The incidence of hilar lymphadenopathy in our study was 0.6%. In the available current literature, only one case report with hilar lymphadenopathy has been reported in COVID-19 [39]. Few studies showed no presence of hilar lymphadenopathy in COVID-19 [21, 40]. Thoracic lymphadenopathy includes hilar and mediastinal group of lymph nodes. Mediastinal lymphadenopathy previously thought to be an atypical feature has been redefined as “not-atypical” feature of COVID-19 [7, 41]. However, hilar lymphadenopathy which is usually associated with fungal infections, mycobacterial infections, and sarcoidosis are seldom seen in COVID pneumonia [39]. Histopathological correlation was unavailable for our cases. Hence, bacterial or fungal co-infection cannot be ruled. Follow-up imaging is to be done to evaluate the persistence or resolution of hilar lymphadenopathy and is required to establish their clinical importance.

**Fig. 7:**
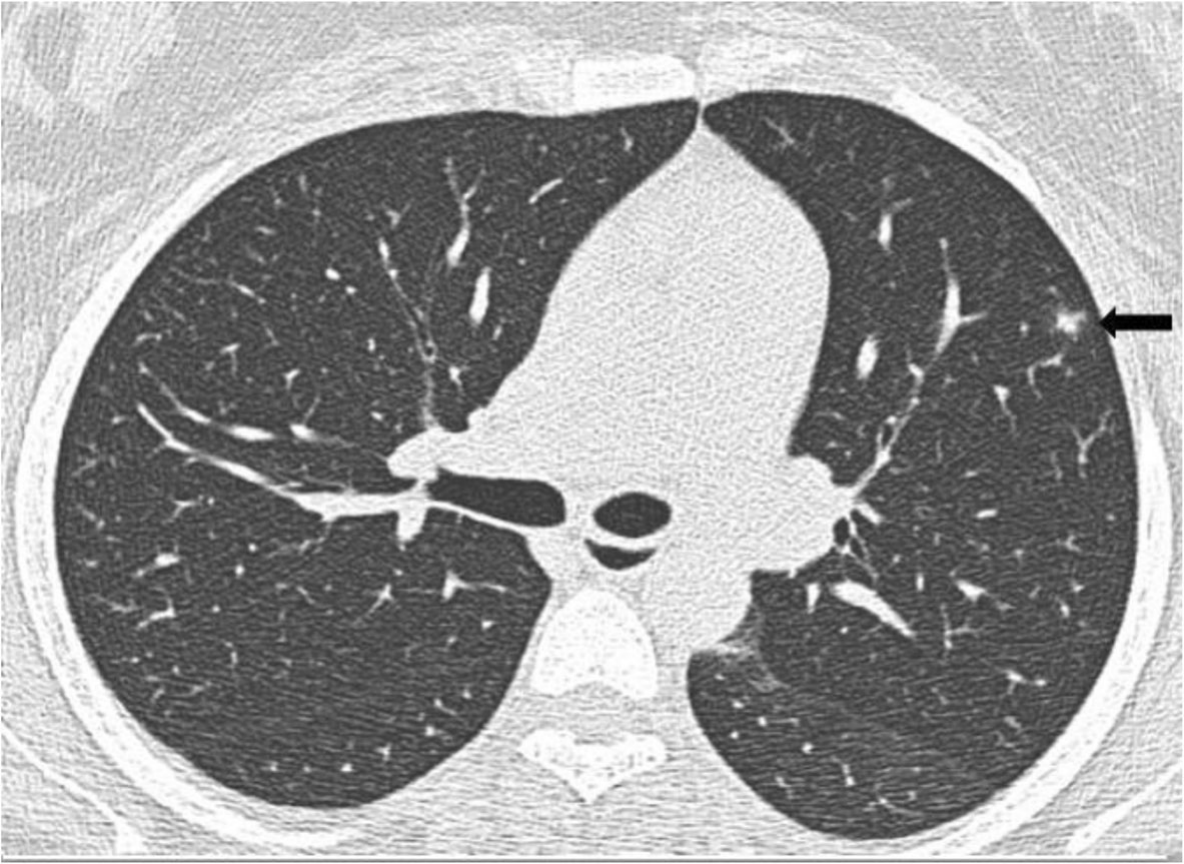
GGO nodule. Axial CT thorax of a 35-year-old COVID pneumonia patient showing small solitary GGO nodule in the left upper lobe as indicated by the arrow

In our study, the incidence of pulmonary nodules was 4.3%. The reported incidence of nodules in COVID-19 has been found to be varying, 3 ~ 13% as per the available literature [42, 43]. The relation between COVID-19 and nodules are not fully understood. Further studies are required to know whether these are incidental nodules or atypical manifestation of COVID-19 pneumonia. Figure 7 shows a case with GGO nodule.

**Fig. 8:**
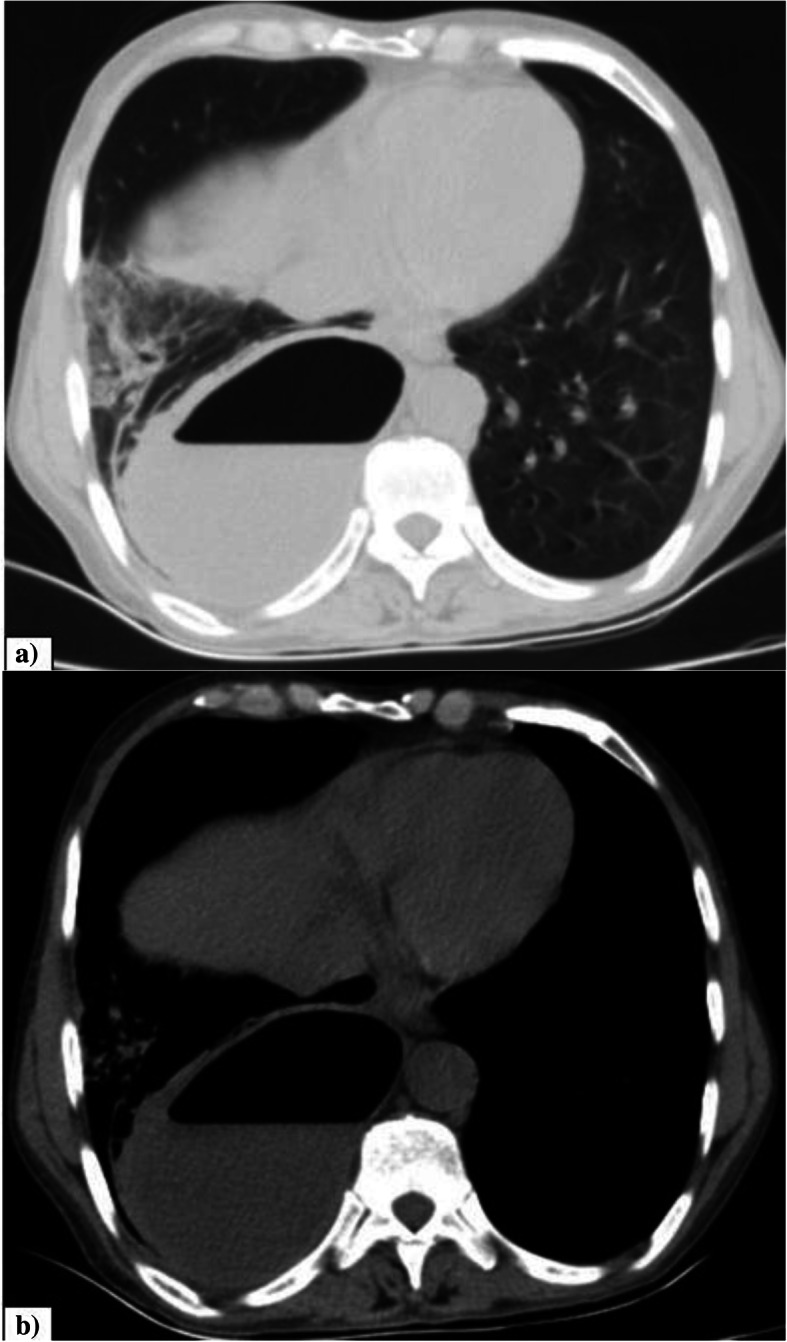
Empyema. **a**, **b** Axial CT thorax (lung window and mediastinal window) of a 63-year-old COVID pneumonia patient showing right sided loculated empyema. Adjacent areas of consolidation are also seen

Empyema has significant clinical morbidity [44]. In our study, a case presented with loculated hydropneumothorax as shown in the Fig. 8. Later, pus-like pleural fluid was aspirated and sent for analysis which showed elevated glucose, protein, and chlorides with predominant neutrophils (90%). This confirms the superadded bacterial infection in COVID-19 pneumonia resulting in empyema. Isolated empyema in COVID-19 is seldom reported in the literature.

**Fig. 9:**
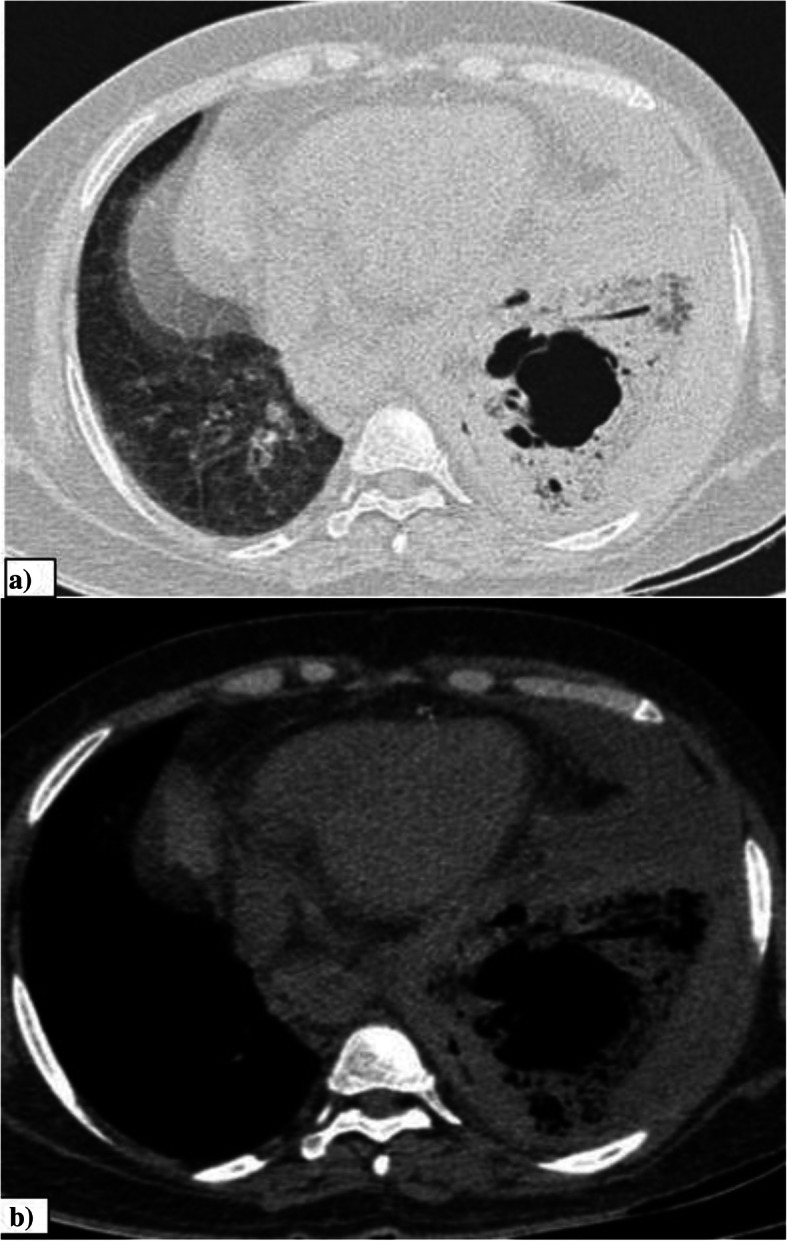
Necrotizing pneumonia. **a**, **b** Axial CT thorax (lung window and mediastinal window) of a 51-year-old COVID pneumonia patient areas of cavitation in the background of consolidation in the left lower lobe. Left pleural effusion also noted

Necrotizing pneumonia is a severe complication of lung infection characterized by necrosis and liquefaction of lung parenchyma, likely secondary to ischemia caused by thrombosis of intrapulmonary vessels [45]. In viral pneumonias, necrotizing processes with development of cavities and air-fluid level in the initial areas of consolidation have been described before in the literature [46]. Similarly, COVID-19 which causes small vessel microthrombi and severe dysregulation of the host immune reaction can result in necrotizing pneumonia. Although bacterial and fungal lung abscesses are known to occur in COVID-19 in up to 11% and 3%, respectively, which were presumed to have formed after hospital admission [47]. Our study showed an incidence of only 0.3%. This discrepancy might be due to the differences in demographic features and hospital care. Figure 9 shows a case of necrotizing pneumonia with secondary cavitation and abscess formation.

## Conclusion

During the course of the pandemic, much of the literature published describes the classical imaging features encountered in COVID-19, with anecdotal references made to the atypical CT imaging features. A small subset of cases with COVID-19 pneumonia show diverse imaging manifestations, which if ignorant can confound the clinical approach to the patient leading to misdiagnosis. The present study aimed not only to illustrate the various atypical CT features in COVID-19 pneumonia but also correlated with disease severity. The atypical features observed includes pulmonary cystic changes, pleural effusion, nodules, bull’s eye/target sign, cavitation, halo sign, hilar lymphadenopathy, spontaneous pneumothorax, spontaneous pneumo-mediastinum, empyema and necrotizing pneumonia with abscess. Significant positive correlation between CT severity score among atypical group and age was observed in our study indicating that with increase in age there was increase in the CT severity score and also of atypical CT features. Thus, a comprehensive knowledge of these atypical pulmonary presentations and its complications on imaging plays an important role in the early diagnosis and management of COVID-19.

## Data Availability

The datasets generated and/or analyzed during the current study are not publicly available due to privacy of the study participants.

## Abbreviations

CT: Computed tomography
Covid-19: Coronavirus disease 2019
GGO: Ground glass opacity
RT-PCR: Reverse-transcriptase polymerase Chain reaction
SARS-CoV-2: Severe acute respiratory syndrome coronavirus 2
PACS: Picture Archiving and Communication Systems
HRCT: High-resolution computed tomography
MDCT: Multidetector computed tomography
SPM: Spontaneous pneumo-mediastinum
